# SARS-CoV-2 Omicron BA.1/BA.2 Neutralization up to 8 Weeks After PrEP With Sotrovimab or Cilgavimab/Tixagevimab

**DOI:** 10.3389/ti.2022.10906

**Published:** 2022-12-12

**Authors:** Karin Stiasny, Lukas Weseslindtner, Andreas Heinzel, Jeremy V. Camp, Rainer Oberbauer, Roman Reindl-Schwaighofer

**Affiliations:** ^1^ Center of Virology, Medical University of Vienna, Vienna, Austria; ^2^ Division of Nephrology and Dialysis, Department of Internal Medicine III, Medical University of Vienna, Vienna, Austria

**Keywords:** COVID-19, kidney transplantation, antiviral, prophylaxis, SARS-CoV2

Dear Editors,

Pre-exposition prophylaxis (PrEP) with monoclonal antibodies (mab) against severe acute respiratory syndrome coronavirus type 2 (SARS-CoV-2) is used to prevent coronavirus disease 2019 (COVID-19) in high-risk individuals with insufficient response to vaccination (1, 2). Currently, cilgavimab/tixagevimab (Evusheld, AstraZeneca) remains the only mab combination approved for PrEP. A significant reduction of *in vitro* neutralization capacity against the Omicron BA.1 variant (B1.1.529) was observed for most mabs including cilgavimab/tixagevimab. A high rate of break-through infections including severe disease following cilgavimab/tixagevimab was observed for BA.1 (3). Sotrovimab (Xevudy, VIR Biotechnology GlaxoSmithKline) retained substantial *in vitro* neutralization capacity against BA.1, and a half-life of 48.8 days made it a candidate for an off-label use as PrEP in high-risk individuals. However, sotrovimab has shown a significantly reduced *in vitro* neutralization capacity against the Omicron BA.2 sub-lineage while cilgavimab/tixagevimab retained strong activity (4).

We used sotrovimab in an off-label indication as PrEP in kidney transplant recipients (KTR) without neutralizing antibodies after at least three COVID-19 vaccine doses at our institution (Medical University of Vienna, Austria) beginning in January 2022 (following the emergence of BA.1), and PrEP was changed to cilgavimab/tixagevimab in March 2022 (following the emergence of BA.2). PrEP was provided to KTR with antibody levels <264 BAU/mL following three vaccinations and no previous history of COVID-19.

In the present analysis, we longitudinally assessed the *in vivo* neutralization capacity of sotrovimab (*n* = 20) against BA.1 as well as BA.2 (proof of principle for sotrovimab as PrEP), and cilgavimab/tixagevimab (*n* = 30) against BA.2 (following the emergence of BA.2) for up to 8 weeks after PrEP (baseline characteristics are provided in Supplementary Table S1). Patients either received 1) 500 mg of sotrovimab intravenously (between January 12 and January 19, 2022) or 2) 300 mg of cilgavimab/tixagevimab by intramuscular injection (between March 4 and March 9, 2022). All patients were followed at the outpatient department of the Division of Nephrology and Dialysis at the Medical University in Vienna (IRB# 1362/2020; 1612/2021). Serum samples were collected at 4 and 8 weeks after antibody administration in all patients as well as 1 hour (only sotrovimab) and 2 weeks (both sotrovimab and cilgavimab/tixagevimab) in a subgroup of patients. Variant-specific live virus neutralization tests (NT) were performed with BA.1 and BA.2 variants. Detailed methods are provided in the supplementary material (5, 6). NT titers of serum samples ≥10 were considered positive. Neutralization titers are reported as median and Q1 and Q3.

All individuals receiving sotrovimab retained neutralization capacity against the BA.1 variant for 4 weeks follow up (FU), and all but one individual still exhibited neutralization capacity against BA.1 at 8 weeks FU. Median NT titers decreased from 30 (Q1, Q3: 30, 40) at 4 weeks to 20 (Q1, Q3: 15, 30) at 8 weeks FU (Figure 1A). In contrast, neutralizing capacity against the BA.2 variant in serum was only present in 60% at 4 weeks and further decreased to 15% at 8 weeks FU. In line, median NT titers against the BA.2 variant were also significantly lower (10 [Q1, Q3: <10, 10] and <10 [Q1, Q3: <10, <10] at 4 weeks and 8 weeks of FU, respectively; Figure 1A). However, analysis in the subgroup with measurements at 1 h and 2 weeks after sotrovimab infusion showed that all individuals had initially achieved neutralization capacity against BA.2 (40 [Q1, Q3: 30, 45] and 20 [Q1, Q3: 19, 30] at 1 h and 2 weeks, respectively).

**FIGURE 1:**
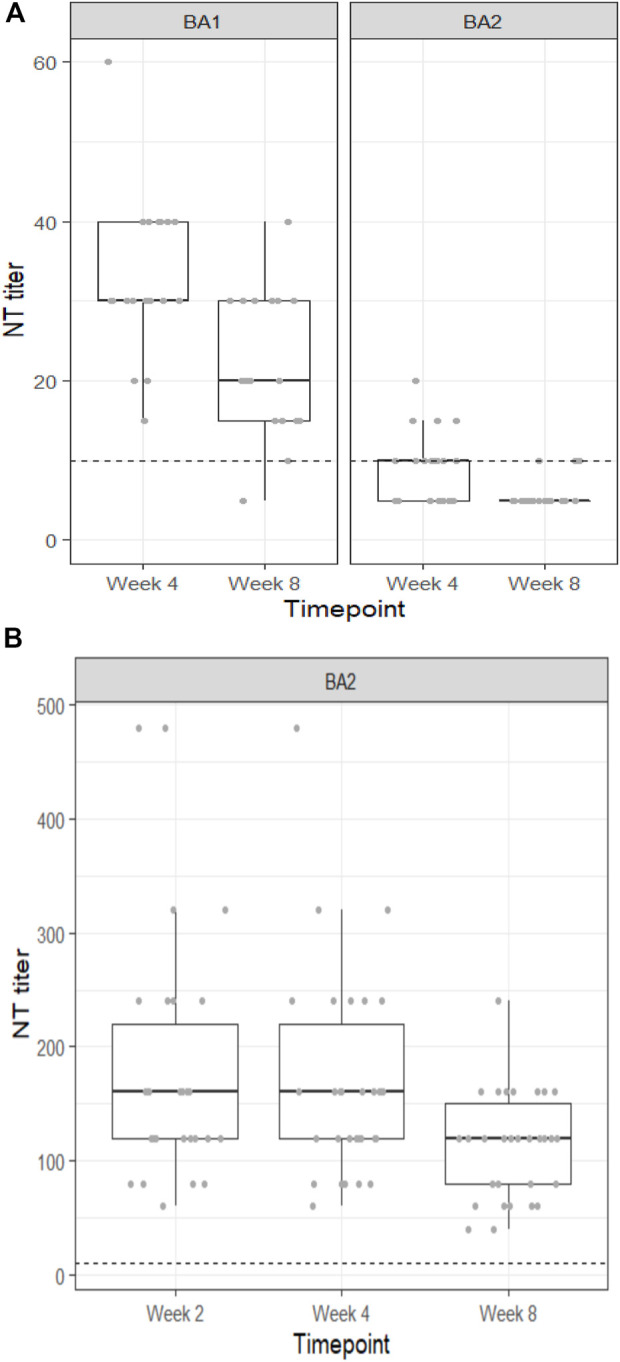
**(A)** Neutralization titers of patient sera against the SARS-CoV-2 Omicron BA.1 and BA.2 variants at four and 8 weeks after prophylactic infusion of sotrovimab. **(B)** Neutralization titers of patient sera against the SARS-CoV-2 Omicron BA.2 variant at 2, 4, and 8 weeks after prophylactic infusion of cilgavimab/tixagevimab.

Patients receiving cilgavimab/tixagevimab had significantly higher neutralization titers against BA.2 than those receiving sotrovimab at all time points: 160 [Q1, Q3: 120, 220], 160 [Q1, Q3: 120, 220] and 120 [Q1, Q3: 80, 150] at 2, 4, and 8 weeks, respectively (Figure 1B).

We could show that sotrovimab retains neutralization capacity against BA.1 for at least 8 weeks while only having a limited neutralization activity against the BA.2 variant. Cilgavimab/tixagevimab on the contrary shows strong *in vivo* neutralization of BA.2 for at least 8 weeks. Our data support that PrEP has to be adapted based on immune-evasion characteristics of pre-dominant SARS-CoV-2 variants.

## Data Availability

The raw data supporting the conclusion of this article will be made available by the authors, without undue reservation.
