# Supplementary material for: SARS-CoV-2 Omicron BA.1/BA.2 Neutralization up to 8 Weeks After PrEP With Sotrovimab or Cilgavimab/Tixagevimab
Source: Transpl Int. 2022 Dec 12;35:10906. doi: 10.3389/ti.2022.10906 (PMC9790897; doi:10.3389/ti.2022.10906)
Supplement: Supplementary file 1 [file DataSheet1.docx]

# Supplementary Material

## Baseline Characteristics

## Table S1: Patient demographics

|  | Measure | |
| --- | --- | --- |
| Parameter | Sotrovimab | Evusheld |
| N | 20 | 30 |
| Age, y *mean (SD)* | 61 (10) | 63 (12) |
| Sex, f *count (%)* | 10 (50%) | 10 (33%) |
| Transplant age, y *median [IQR]* | 6 [4 - 9] | 6 [3 – 9] |
| Spike Ab, U/mL *gmean (GSD)* | 0.43 (4.93) | 2.03 (12.07) |

## Neutralization assay

The live virus neutralization test (NT) was performed as described previously (1, 2). Briefly, two-fold serial dilutions of heat-inactivated serum samples or monoclonal antibodies (mabs, sotrovimab or Evusheld) were incubated with 50–100 TCID50 SARS-CoV-2 (Omicron BA.1: EPI_ISL_9110894, Omicron BA.2: EPI_ISL_11110193) for one hour at 37°C before the mixtures were added to Vero E6 cells (ECACC 85020206). Sera were tested in duplicates; 10 replicates were used for each mab dilution. After five days at 37°C, NT titers were expressed as the reciprocal of the serum dilution required for prevention of virus-induced cytopathic effects. NT titers of serum samples ≥10 were considered positive.

IC50 (50% inhibitory concentration) and IC90 (90% inhibitory concentration) values for sotrovimab and Evusheld were determined by non-linear regression analysis (four-parameter logistic curves) with GraphPad Prism 9.4.1 and are depicted in Figure S1 and summarized in Table S2.

## Figure S1

Neutralization of Omicron variants by monoclonal antibodies (mab). Geometric mean values of mab concentration to inhibit infectivity by 50% (IC50) and 90% (IC90) are from 10 (Sotrovimab, BA.1), 9 (Sotrovimab, BA.2) and 15 (Evusheld, BA.2) independent experiments. Error bars indicate 95% confidence intervals. (a and b) Neutralization of Omicron BA.1 and BA.2 by Sotrovimab. (c) Neutralization of Omicron BA.2 by Evusheld.


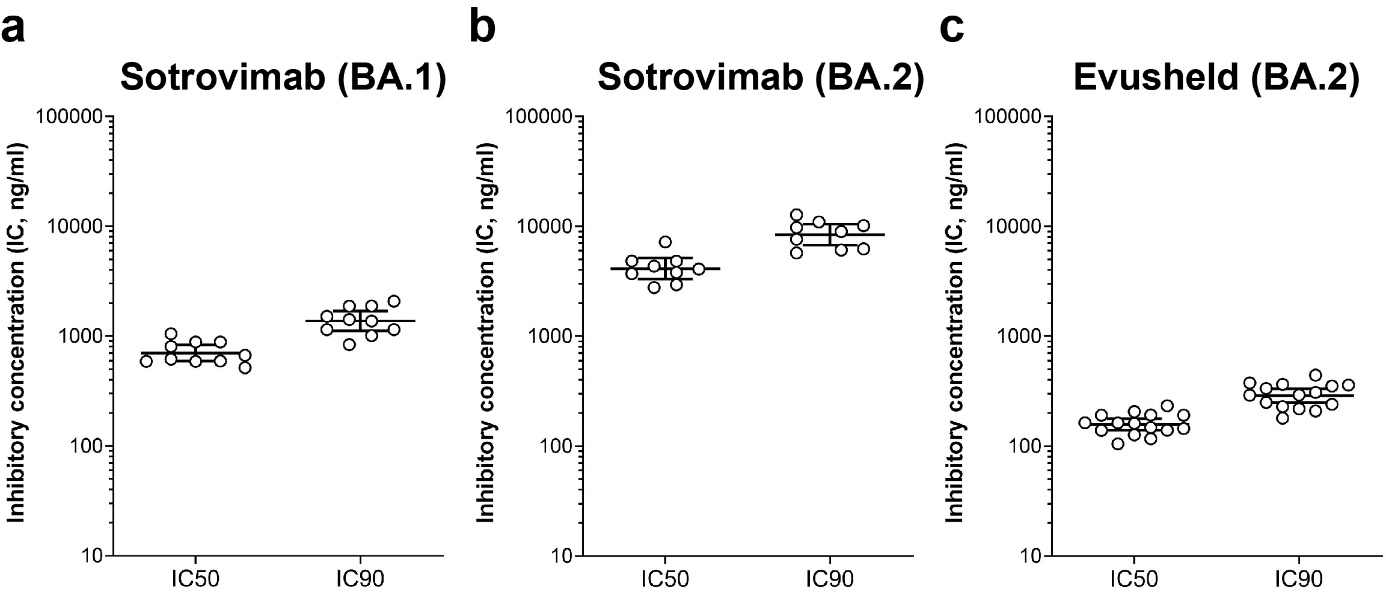


## Table S2

IC50 and IC90 concentration of monoclonal antibodies (mab) against Omicron variants.

|  | **Sotrovimab** | | | | **Evusheld** | |
| --- | --- | --- | --- | --- | --- | --- |
|  | **BA.1** | | **BA.2** | | | |
| N | 10 | 10 | 9 | 9 | 15 | 15 |
|  | *IC50* | *IC90* | *IC50* | *IC90* | *IC50* | *IC90* |
| **GM (ng/ml)** | **702.4** | **1375** | **4130** | **8397** | **157.8** | **288** |
| 95% CI | 594.3 | 1114 | 3314 | 6740 | 139.6 | 249.6 |
|  | 830.1 | 1698 | 5147 | 10461 | 178.4 | 332.4 |

N = number of independent experiments
GM = geometric mean
CI = confidence interval

## References

1. Sieber J, Mayer M, Schmidthaler K, Kopanja S, Camp JV, Popovitsch A, et al. Long-Lived Immunity in Sars-Cov-2-Recovered Children and Its Neutralizing Capacity against Omicron. Frontiers in Immunology (2022) 13. doi: 10.3389/fimmu.2022.882456.
2. Medits I, Springer DN, Graninger M, Camp JV, Höltl E, Aberle SW, et al. Different Neutralization Profiles after Primary Sars-Cov-2 Omicron BA.1 and BA.2 Infections. Frontiers in Immunology (2022) 13. doi: 10.3389/fimmu.2022.946318.

## Acknowledgements

We thank Jutta Hutecek and Silvia Schwödiauer for excellent technical assistance.
